# Self-care interventions of community-dwelling older adults: a systematic review and meta-analysis

**DOI:** 10.3389/fpubh.2023.1254172

**Published:** 2023-10-09

**Authors:** Estela González-González, Carmen Requena

**Affiliations:** Catedra de Envejecimiento en todas las edades, University of León, León, Spain

**Keywords:** self-care, healthy older adults, community-dwelling, interventions, health

## Abstract

**Introduction:**

The current notion of “care in old age” should be reconceptualized in the ageing societies of the 21st century. Currently, “being old” means that one is actively involved in their care and has the desire to retain control and independence.

**Objective:**

Understand and analyze the efficacy of interventions in the physical and psychological self-care practices of healthy community-dwelling older people.

**Methodology:**

Systematic review and meta-analysis. The guidelines of the PRISMA guide were followed. The methodological quality of the studies was checked using Cochrane Effective Practice and Organisation of Care criteria, and the search was performed between 2016 and 2021.

**Results:**

Of the 1,866 evaluated, 8 studies met the criteria. The systematic review reveals that self-care interventions focus on physical health-related variables but not on psychological variables. The meta-analysis shows that interventions significantly improve physical health-related variables (care visits, hospital admission, medication, and gait speed).

**Conclusion:**

Self-care training programs should include psychological variables to increase health and well-being in healthy older people.

## 1. Introduction

The chance that older people currently have of living longer is made in better bio-psych-social conditions, with a higher educational level and better-qualified work performance that sexagenarians from as recently as one decade ago (1). Moreover, today’s older adults (or pre-older adults) population practice healthier lifestyles, have good social networks and freely choose how to spend their free time (2). Attending to the new profile of older people in ageing 21st century societies necessitate reconceptualizing the current notion that “being old” means that one is a passive subject needing care. This proactive ageing approach is characterized by considering that ageing is not only the result of the curse of time in genetic determinants but conditioned by social and environmental factors. Furthermore, these social and environmental factors could get to determine the course of ageing up to 75% (3). Therefore, more than just genetic factors or hereditary conditions on age and sex are involved in the sociopolitical decisions that determine the independent functionality of older people until the end of their lives. Hence, the real challenge of ageing research is to eradicate the biased medical vision of ageing as an inevitable period of loss leading to dependency and disability and replace it with a multidimensional model that predicts personal development in ageing through self-care practices (4).

Classically, self-care has been studied from the unidisciplinary approaches of nursing and is defined as the set of all those human physical, mental, and psychological practices aimed at maintaining and/or restoring the health and well-being of people in the community (5). The main life-cycle theories underlying this health approach advocate that adulthood is a largely period of loss mitigation and prioritization of goals related to maintaining functional independence (ability to perform daily tasks) over the long term (6). In this framework, self-care education programs for older adults promote daily self-management of chronic conditions. In general, chronic diseases (e.g., hypertension or diabetes) cannot be cured, but symptoms can be controlled with lifestyle changes (7). Also, these educational programs propose practices to prevent and treat diseases such as Alzheimer’s (e.g., cognitive stimulation programs or memory training) (8).

While loss mitigation is an important goal, we argue that it is equally necessary to investigate how older adults can improve their current state, especially among those who do not experience functional or cognitive deficits. Prominent life course theories propose that the core of self-care is not only about preserving health as the absence of disease but also includes practices that stimulate life and optimal personal development (9). This conceptualization of self-care is more in line with the WHO model of healthy ageing (10), which focuses on the practices of activities that enhance a person’s physical and mental capacities. It is also biopsychosocial and developmental approaches that emphasize the concept of resilience, neuroplasticity, and people’s continuous capacity to adapt and develop throughout life (11).

One should consider that one of the demands of the new older adults has to do with the challenge of self-care in their own home. Consequently, it is essential to provide a socio-community intervention plan for good self-care practices. These types of practices should consider not only a care network of family, friends, and institutions on which one can count but also, especially, a psychoeducational plan of personal development in the long term (12). A psychoeducational plan of personal development focuses on adapting for growth by learning new skills to prepare older people to handle any changes that may emerge over time. Therefore, new social healthcare policies must respond to new social and psychological claims, in addition to the health promotion that contributes to maintaining a full life in one’s own home.

So far, some systematic reviews and meta-analyses have examined the effect of specific behavioral interventions for the care of older adults with long-term conditions (LTCs) (13), such as frailty (14) or sarcopenia (15). However, as far as the authors know, there are no studies that synthesize the findings in a comprehensive range of behavioral interventions for self-care that target healthy community-living older adults. Behavioral interventions focus on training in daily living practices related to behaviors that optimize physical and psychological health. Unlike other behavioral interventions, they are not focused on the management of chronic diseases. This divide between interventions for LTCs and interventions for optimizing health is addressed by examining how studies include intervention in daily living care programs and what interventions, if any, provided such benefits to the target population. It is important to highlight that this review will emphasize the comparisons between classic interventions, which treat older people as “the passive subject of care,” and interventions based on the proactive model of ageing, which advocates for a caregiving-engaged older person profile. Moreover, following the Cochrane Effective Practice and Organisation of Care Review Group quality criteria for reviews (16), essential comparisons within and between interventions are considered, as there may be some intervention types with similar strengths in the same trial interventions (i.e., active control groups adequate) or with systematic weakness in the trials (i.e., inadequate boosts). Furthermore, this review will identify which indicators are better for promoting and maintaining older adults’ self-care habits/practices for those who want to stay home. It will select the details of the intervention that are evaluated in the transfer, such as duration and dose, and it will consider the follow-up period. Ultimately, this systematic review and meta-analysis specifically pursue: (1) summarizing similarities and differences between different care intervention programs and their results, (2) identifying care intervention programs that grant benefits in cognitive, psychological, and physical domains, and (3) highlighting the limitations that should be addressed in future research.

## 2. Methods

### 2.1. Rationale

This systematic review and meta-analysis only considered randomized controlled trials (RCTs) that were aimed at supporting self-care in community-dwelling older adults. Participants of the intervention are distributed into two groups: an intervention group, which received psychoeducational self-care interventions, and a control group, which received usual care (not receive psychoeducational self-care structured programs). The definition of self-care used in this review was associated with care practices in which older people decide not only to promote or maintain health and functioning in ageing but also how to optimize it. Studies that focused on disease-specific self-care were outside the scope of this review since these programs involved disease-specific skill-based training, and the support is often delivered after hospital discharge.

### 2.2. Inclusion and exclusion criteria

Only articles from peer-reviewed journals published in English with a focus on healthy community-dwelling older adults (60+ years) and from any country were included. Included studies reported the outcome of a behavioral intervention on physical and psychological health. Papers based on the same study sample were included. The PICO framework used to define the eligibility criteria is seen below:

P—Population: healthy and community-dwelling older adults 60 years of age and older.
I—Intervention: psychoeducational self-care interventions.
C—Comparison: usual care.
O—Outcome: physical and psychological health.

Only RCTs on psychoeducational (behavioral) self-care interventions were eligible (see Table 1). Studies involving people under 60 years, people with dementia diagnosis, physical impairment, or long-term conditions (LTC), and pharmaceutical interventions were excluded. The search items can be seen in Table 2.

**Table 1:** Inclusion/exclusion criteria.

	Inclusion	Exclusion	Rationale
Population	Healthy and community-dwelling older adults 60 years of age and older	People under 60 years or older people with serious diseases	To review self-care activities in healthy community older adults
Intervention	Behavioral self-care intervention	Pharmaceutical or disease management intervention	To focus on self-care interventions
Comparison	Usual care		
Outocome	Health and wellbeing benefits	Improvement of the disease or compensatory strategies	To investigate benefits on physical and psychological health

**Table 2:** Search items.

Care (step 1)		Context (step 2)		Population (step 3)
Terms	AND	Terms	AND	Terms
“care” OR “take care” OR “self-care” OR “care of the self” OR “be cared for”		“healthy” OR “health” OR “healthy ageing” OR “healthy ageing” OR “community-dwelling”		“older age” OR “older people” OR “older adult” OR “ageing” OR “ageing”
**Combined with OR**		**Combined with OR**		**Combined with OR**

### 2.3. Search strategy

The review was conducted in PubMed, Medline, Scopus, Web of Science (WoS) and Cochrane Library databases. Keywords for the type of care intervention, context, and population were used to search each database. The terms can be seen in the database search history (Supplementary material B).

**Figure 1:**
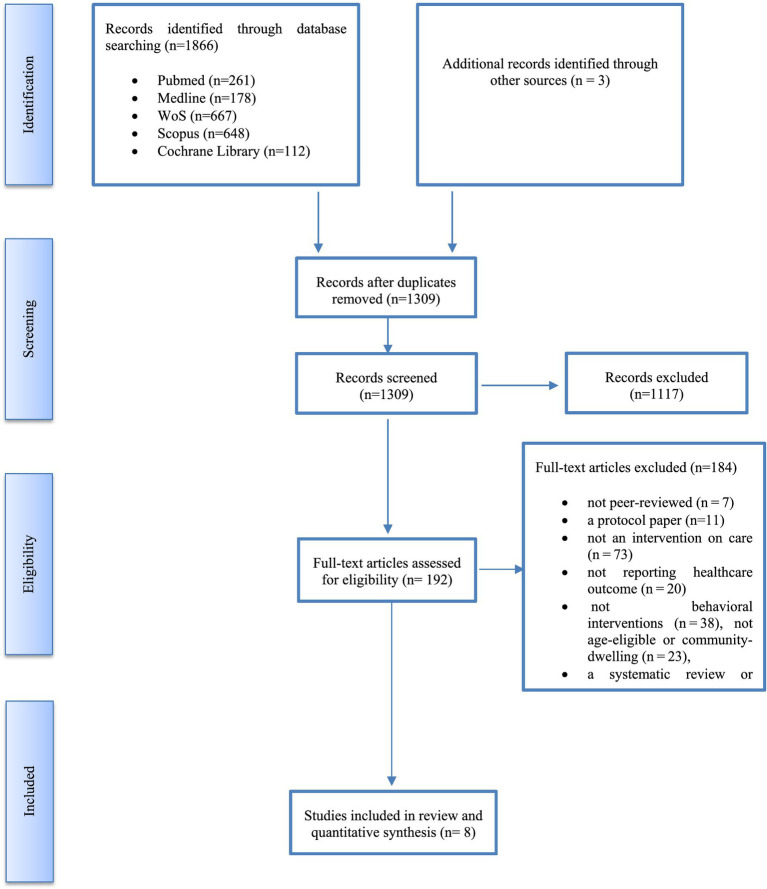
Search result (PRISMA).

The key categories searched were care (self-care), healthy community-dwelling, and older adults. Synonyms for each key category generated were individually searched and then collectively combined with the “OR” logical operator. An intersection between the three key categories searched was done using the “AND” operator as shown in Table 2. The screening process from the five databases yielded 1866 total hits and is presented in the PRISMA flow diagram in Figure 1 [Pubmed (*n* = 261 articles), Medline (*n* = 178 articles), Scopus (*n* = 648 articles), the Wos (*n* = 667 articles) and the Cochrane Library (*n* = 112 articles)]. Duplicated articles were excluded (*n* = 557). Across both search strategies, a total of 1,309 articles were screened, and 1,117 articles were excluded based on title and abstract. The remaining 192 articles were screened in full. Of those, 184 were excluded. Excluded studies were: not peer-reviewed (*n* = 7), protocol papers with no reported results (*n* = 11), not focused on an intervention on care (*n* = 73), not reporting healthcare outcome measures (*n* = 20), not behavioral interventions with posttest measures (*n* = 38), not age-eligible or did not include community-dwelling older adults (*n* = 23), or a systematic review or meta-analysis that was pulled for cross-check purposes only (*n* = 12). Finally, 8 unique articles referring to a total of 7 studies were included in the current systematic review (Figure 1).

### 2.4. Data extraction and quality assessment

Two reviewers (CR and E-GG) independently reviewed articles in-depth and extracted data independently based on the inclusion criteria. Each article was crosschecked by both authors, and any discrepancies were resolved through mutual discussion to achieve consensus.

Data from the eight selected studies were extracted under these headings: name of the author (s), year of publication, study design, methods, and intervention. Methodological quality was assessed using the Cochrane Effective Practice and Organisation of Care Review Group quality criteria for reviews (16). These quality criteria evaluate the risk of bias in each intervention. Specifically, this validated methodology is composed of nine criteria (Random sequence generation, Allocation concealment, Baseline outcome measurements similar, Baseline characteristics similar, Incomplete outcome data, Knowledge of the allocated interventions adequately prevented during the study, Protection against contamination, Selective outcome reporting, and Other risks of bias) with the aim of summarizing the evidence to guide health system decision-making to improve socio and health services and population health outcomes. If information was omitted from the article, reviewers are referred to protocol papers and ClinicalTrials.gov or other trial registration platforms. Each item was scored either: low risk of bias, unclear risk of bias, or high risk of bias. An outcome was considered to have a “low risk of bias” if the study showed a low risk of bias in all key domains, “unclear risk of bias” if the risk of bias was unclear for one or more key domains, and “high risk of bias” if there was a high risk of bias in one or more key domains (17).

### 2.5. Statistical analysis

Meta-analyses were conducted using Review Manager (version 5.4). We decided to use fixed effects meta-analysis, considering the size of the study and its own variance as the only determinants of its weight. The standardized mean differences (SMDs) and their 95% confidence intervals (CIs) were calculated from post-intervention outcomes for continuous data, while the odds ratio (OR) was obtained from dichotomous data. Pooled ORs (95% CI) were calculated, and a two-sided *p*-value <0.1 was considered to indicate statistical significance (18).

## 3. Results

### 3.1. Study characteristics

Of the 8 studies included in this study, 2 were related to the same research (19, 20). Among the 8 studies, 1,798 participants between 31 (21) and 540 participants (22) were included, with an average sample size of 257 participants per study. The average age was 75.21 years, ranging between 60 and 92 years. On the other hand, the female gender had a representation average in the studies of 64.9% of the sample, ranging from 51.6% (21) to 75.1% (19, 20). The Wong et al. (22) study does not identify gender or age.

Studies used various channels, visit numbers, durations, and providers to carry out the care interventions in community-living older adults. Regarding delivery channels, 2 (25%) trials used only home visits, 3 (37.5%) used home visits and a telephone follow-up, 1 (12.5%) used home visits and group training, and 1 (12.5%) used visits to a community center. Two care program types were identified: intensive (care duration longer than 6 months) (6 studies) and intermediate (care duration between 6 months to 1 year) (2 studies). None of the included studies was of the extensive type (care duration longer than 1 year). Participants received an average of 20.66 sessions, ranging from 8 to 48, with an average duration of 62 min, ranging between 30 and 90. One study (23) did not specify the number of sessions carried out in the intervention. Regarding care program providers, three 3 of them were from a single discipline [two from nursing (22, 24) and one from occupational therapy (25)], 3 were multidisciplinary by socio-health care providers [two combined nurses with social and community workers (19, 20), and one was performed by a team composed by physical therapists and educators (26)]. Finally, two articles did not specify who the interventions providers were Dolovich et al. (23) only refers to volunteers, and Wong et al. (20) is online. The protocol was not found in 1 study (24). A summary of study characteristics can be seen in Table 3.

**Table 3:** Characteristics of the included studies.

				Intervention						
Study	Sample	Age	Gender	Chanel	Visit	Duration	Provider	Approach	CG	Results
18	457 (IG = 230; CG = 227)	78 (±7.92)	75.1% females	Home and telephone calls	8 session (3 month)	First: 1 h. Next: 1 h. Telep.: 20 m.	Nurse and social and community workes	Classic	Usual care	Improve psychological health
19	457 (IG = 230; CG = 227)	78 (±7.92)	75.1% females	Home and telephone calls	8 session (3 month)	First: 1 h. Next: 1 h. Telep.: 20 m.	Nurse and social and community workes	Classic	Usual care	Improves psychological and physical health
20	31 (IG = 15; CG = 16)	73.9 (± 6.4)	61.6% females	Home and telephone calls	48session (4 month)	30–40 m.	Online	Classic	Usual care	Improve cognitive, not brain health
21	540 (IG = 271; CG = 269)	–	–	Home and telephone calls	8 session (3 month)	First: 1 h. Next: 1 h. Telep.: 20 m.	Nurse	Classic	Usual care	Reducing service cost. Program cost effective.
22	312 (IG = 158; CG = 154)	IG = 78.06 (± 6.3) CG = 79.06 (± 6.6)	62.2% females	Home	6 month	–	Volunteers	Classic	Usual care	More care visit, fewer hospital admission
23	136 (IG = 69; CG = 67)		58.8% females	Local venue and home	8 session (2 month) + 8 follow session	1 h/session +30 m/day follow	Nurse	Classic	Not education program	Improves healthy lifestyle
24	262 (IG = 136; CG = 126)	IG = 72.9 CG = 71.3	74.8% females	Local venue	20session (4 month)	–	Ocupattional therapy	Classic	Usual care	Intervention not effective for UK
25	60 (IG = 30; CG = 30)	IG = 74 (±4.9)	66.6% females	University	24session (6 month)	90 m.	Physic therapist and educators	Classic	Didactic teaching method	Improves healthy lifestyle

IG, intervention group; CG, control group; h, hour; m, minute.

### 3.2. Methodological quality of the studies

Agreement between two independent reviewers was greater than 90% in all aspects of the quality assessment of the methodology. The quality of the studies was heterogeneous, although the majority have a “high” risk of bias. All the studies adequately described the random sequence generation. Moreover, the great majority informed the Allocation concealment in the correct form, except for the Tavakkoli Oskuei et al. study (24), which may have led to a selection bias. Moreover, the totality of the studies showed a “low” risk of bias for the “selective outcome reporting” criterion.

On the other hand, a great risk of bias [either “high” (18, 19, 21) or “unclear” (21, 23–25)] is obtained in the indicator of baseline characteristics similar, because the baseline characteristics of the intervention and control providers were either correctly reported or not similar. At least half of the studies presented an “unclear” or “high” risk of bias for the baseline similar outcome measurements, to the knowledge of the allocated interventions or other risk criteria.

Finally, regarding the general assessment of the studies’ risk of bias, no publication achieved a “low” risk of bias, 2 studies obtained an “unclear” risk of bias (21, 25), and the 5 remaining studies obtained a high score in the risk of bias. The assessment of the methodological quality of the studies can be seen in Table 4.

**Table 4:** Evaluation of methodological quality by Cochrane criteria.

Study	Random sequence generation	Allocation concealment	Baseline outcome measurements similar	Baseline characteristics similar	Incomplete outcome data	Knowledge of the allocated interventions	Protection against contamination	Selective outcome reporting	Other risks	Total
18	Low	Low	Low	High	High	Low	Low	Low	Unclear	High
19	Low	Low	High	High	High	Unclear	Low	Low	Unclear	High
20	Low	Low	Unclear	Unclear	Low	Unclear	Low	Low	Low	Unclear
21	Low	Low	Unclear	High	Low	Low	Low	Low	Unclear	High
22	Low	Low	Low	Unclear	Low	High	Low	Low	High	High
23	Low	Unclear	High	Unclear	Low	High	Low	Low	High	High
24	Low	Low	Low	Unclear	Low	Low	Low	Low	Low	Unclear
25	Low	Low	Low	Low	Low	Low	High	Low	Low	High

### 3.3. Quantitative synthesis

In the quantitative synthesis are two separate sections: sanitary and psychological aspects. Each section describes the result of the meta-analyses of some sanitary and psychological variables.

#### 3.3.1. Sanitary aspect

Care visit. This variable includes 3 studies (43%) with a total of 1,228 participants. For 3 months, data seem to show a trend of greater health service utilization on the part of the control group (I^2^ = 90%; DME = −0.06; IC of 95% = −0.18, 0.05; *p* = 0.27), although the differences were not statistically significant. At 6 months, the trend of fewer care visit by the intervention group continued keeping (I^2^ = 92%; DME = −0.10; IC of 95% = −0.22, 0.01; *p* = 0.07).

Hospital admission. This variable includes the same studies as the care visit variable. For 3 months, a common significant effect of minor recourse utilization on the part of the intervention group was observed (I^2^ = 0%; DME = −0.35; IC of 95% = −0.35, −0.08; *p* = 0.002). At 6 months, the same trend of fewer hospital admission by intervention group continued keeping (I^2^ = 0%; DME = −0.21; IC of 95% = −0.34, −0.07; *p* = 003).

Medication. This variable includes 2 studies (29%) with a total of 769 participants. Data for the intervention group show a significant trend of better medication intake (I^2^ = 65%; DME = −0.31; IC of 95% = −0.57, −0.04; *p* = 0.03).

Gait speed. This variable includes 2 studies (29%) with a total of 91 participants. Data for the intervention group seem to show an upward trend in gait speed (I^2^ = 0%; DME = −0.09; IC del 95% = −0.18, 0.01; *p* = 0.06), but the difference is not significant among groups.

**Figure 2:**
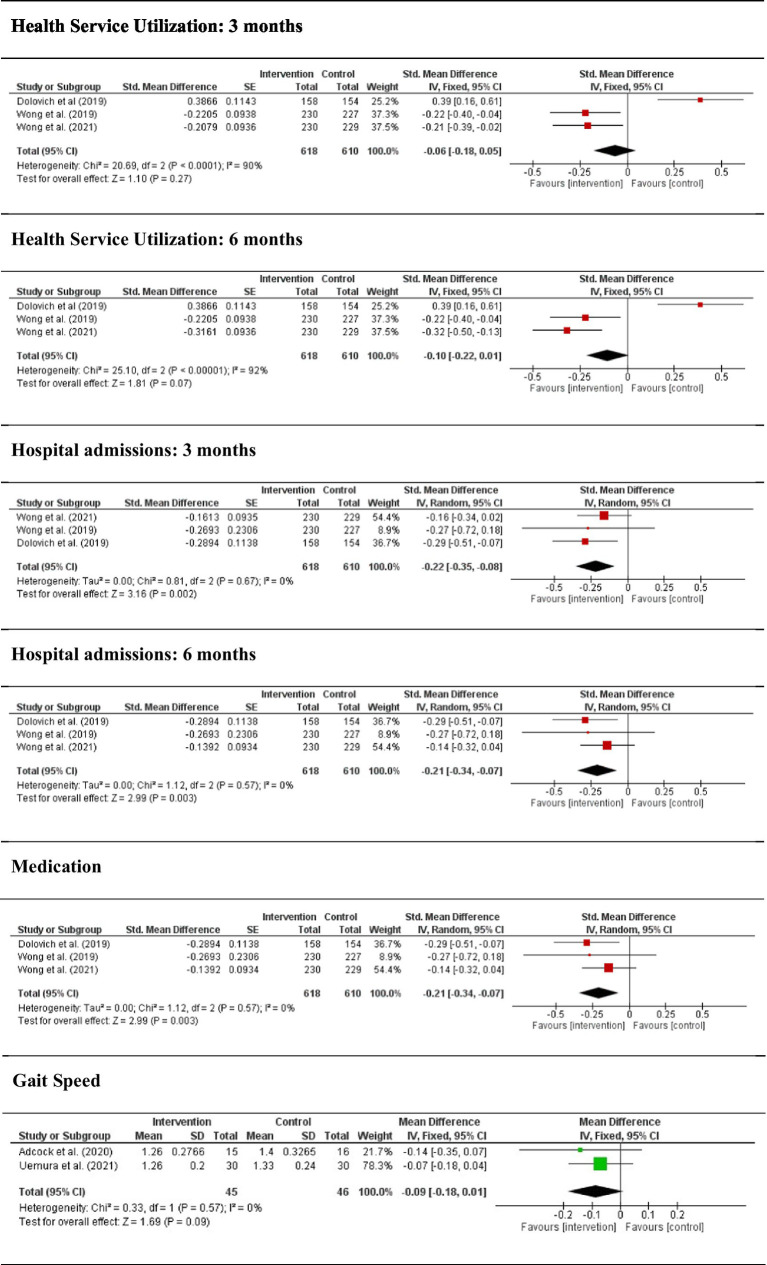
Forest plot between self-care intervention and control group in sanitary aspects.

Figure 2 shows the forest plot of sanitary variables analyzed.

#### 3.3.2. Psychological aspect

Self-efficacy. This variable includes 2 studies (29%) with a total of 769 participants. Data do not seem to show any trend in the improvement of self-efficacy (I^2^ = 0%; DME = 0.00; IC of 95% = −0.013, 0.13; *p* = 0.98), and there are no significant differences between groups.

Quality of life, psychological component. This variable includes 4 studies (57%), with a total of 1.571 participants. Data for the control group seem to show a trend of great quality life (I^2^ = 76%; DME = 0.11; IC of 95% = 0.01, 0.22; *p* = 0.03).

Depression. This variable included 2 studies (29%) with a total of 769 participants. Data for the control group seem to show a tendency of great depression (I^2^ = 0%; DME = 0.05; IC of 95% = −0.06, 0.17; *p* = 0.35), but significant differences do not exist among groups; consequently, it is not possible to establish any general conclusion.

**Figure 3:**
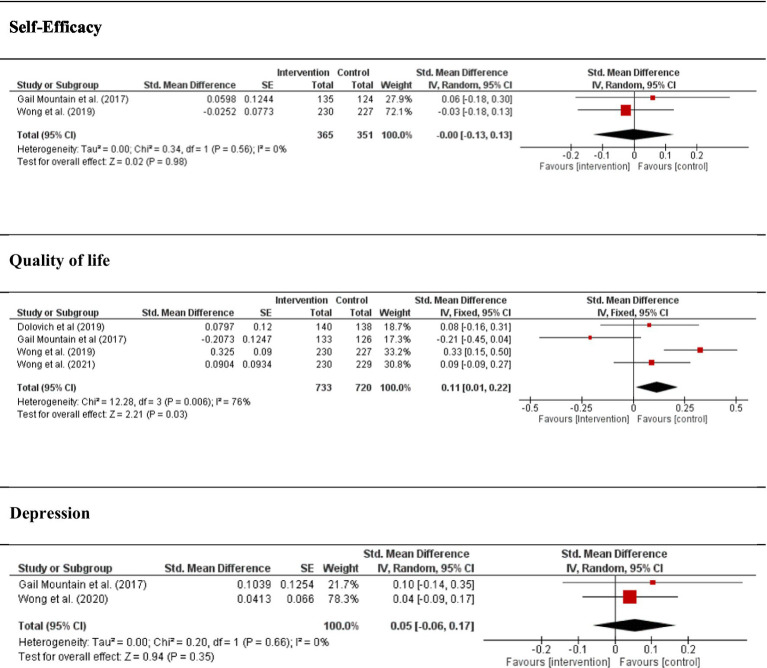
Forest plot between self-care intervention and control group in psychological aspects.

Figure 3 shows the forest plot of psychological variables analyzed.

## 4. Discussion

As far as the authors know, this is the first study on systematic review and meta-analysis of self-care interventions carried out in the community with a healthy population that did not have any diseases or disabilities. Results prove that in addition to there being scarce scientific literature on self-care interventions in healthy older adults, the studies included in this review present a high risk of bias in some criteria of the Cochrane Effective Practice and Organisation of Care Review Group related to a characteristics and outcomes baseline. Nevertheless, the revised interventions reveal that these types of programs are potentially effective and beneficial in the long term with respect to decreasing hospital admissions and show a lower trend to request medical appointments. Moreover, good practices are observed in compliance with prescribed medication intake, and improved agility in physical movement. No effects of these programs on the psychological component of quality of life and self-efficacy, but it does influence mood, although this effect is the opposite of the expected one. Nor has it been possible to establish doses or amounts of normative training.

Results of the systematic review revealed that the contents of the intervention focus on health variables associated with disease, regardless of whether the context of application is health or social field (see Supplementary material A) (27). Although interventions have a preventative purpose, they are based on classic ageing models and the themes topics focus on how to cope with inevitably age-related limitations or declines (28). Interventions are provided by health staff, principally nurses, and are sometimes supported by social works (20, 22). In particular, the variables addressed in the intervention programs are essentially physical health variables such as nutrition or physical exercise (see Supplementary material A). These data could be explained on the basis that older people who do not have healthy behaviors either overuse health and social care resources or need more frequent medical care due to a lack of good self-care practices (29). Older people who present unfavorable measures in impairment and cognition, *inter alia*, tend to use less preventative health services (30), which increases the likelihood of institutionalization and healthcare cost (31). Furthermore, some studies show that older adults who do not practice self-care tend to suffer from a greater variety of psychological disorders more frequently (32). In contrast, when people are engaged with their physical and psychological health, they have a higher probability of maintaining their emotional independence, improving their perception of their quality of life, and having greater control over their negative responses (33).

The sample of this study was mainly females (around 65%) with an average age of 75 years, hence they belong to the classification of “ageing” group (34). Usually, in research with older populations, the profile of greater participation of females is repeated, as well as the practice of forming samples with both males and females, but in the analysis of the results no studies have been found that disaggregate data by sex (35). However, the topic of “self-care” needs specific programs for men and women due to the cultural tradition of associating care practice with females as opposed to males (36). That is for older females, care is continuous, while for males, care is an essential role who must learn in the end of their lives.

For its part, the ageing profile by which self-care programs are inspired is biased toward decline and losses (see Table 1). Furthermore, considering the Baltes lifespan Model, intervention proposals underscore how to carry out environmental or personal compensation for future losses (6). In this sense, some studies propose care interventions in the clinical-specific context of ageing, despite having verified that these types of programs have a greater efficacy when they are applied in an informal psychoeducational context, in which psychological topics related to vital well-being and personal growth (37). However, another more optimistic perspective on development in ageing is possible, as proposed by Nguyen et al. (6). Some experts propose swapping the image of older people as passive agents of care who live in static environments for a realistic and more adaptive model based on the new “ageing” who want to stay home and engage with the community (38). Consequently, health must be understood from a more holistic and comprehensive perspective, taking into account forms of self-care interventions that have not only physical health content, but also long-term life plans that involve challenges of personal development. Moreover, an interdisciplinary approach that addresses psychological, sanitary, environmental and designs that contribute to optimal care until the end of one’s life is needed (39).

Confidence in self-care behaviors is one of the key factors that determines adherence and compliance to self-care (see Supplementary material). Feeling confident in one’s own ability to adequately handle the stressors of daily life refers to the concept of self-efficacy expectation (40). In general, older people know that certain actions such as exercising, taking classes, or reuniting with their peer group benefit their health and well-being (41). In this sense, some studies have demonstrated that learning good care practices and training on the interventions can have a sustained effect on self-efficacy; that is to say, the confidence one has in one’s ability to manage one’s own health (42). Even more, in practice the action, older people who feel effective choose more challenging tasks, set high goals and are more persistent in achieving them (43). Nevertheless, no research has been found that can provide clarity certainly as to whether self-efficacy in self-care behaviors can last in the long term after interventions cease (44).

This study presents some limitations related to bias that affects methodological quality, the type and sample size and specifically with the emerging state of care which is the central topic of this research. However, despite these limitations, this review shines light on the need for promoting self-care programs for healthy older adults without disabilities who live and want to continue living in their houses. That is, actions are needed not only to promote physical health, but also good self-care practices aimed at fostering an optimal state of personal social and psychological growth.

## 5. Conclusion

The research conducted shows that self-care practices intervention promotes the physical health of healthy older people. However, although these programs implement psychological variables in the evaluation of the effect, these are not considered in the training phase. Future studies that implement multidimensional components are needed to understand the real scope of these interventions.

## Author contributions

EG-G: Writing – original draft, Writing – review & editing. CR: Writing – original draft, Writing – review & editing.
