# Supplementary material for: Self-care interventions of community-dwelling older adults: a systematic review and meta-analysis
Source: Front Public Health. 2023 Oct 9;11:1254172. doi: 10.3389/fpubh.2023.1254172 (PMC10593480; doi:10.3389/fpubh.2023.1254172)
Supplement: Supplementary file 1 [file Data_Sheet_1.PDF]

Supplementary Material

1 Supplementary Material A.

Table 1. Characteristics of the self-care interventions for healthy older adults.

Study	Purpose	Contents	Measurement
18	Supportive Care	Promoting physical and mental health<br>Environmental, psychosocial, physiological and health-related behavioural problems<br>Goals of the care plan	Geriatric Depression Scale<br>Life satisfaction<br>Short Form Health Survey, version 2.
19	Supportive Care	Mastery experience<br>Vicarious experience<br>Social and verbal persuasion<br>Physiological and affective states<br>Problem classification scheme<br>Intervention scheme<br>Problem ration scale for outcomes<br>Routines<br>Information systems<br>Meeting<br>Boundary spanner	Chinese version Mini-Mental Status Examination<br>The General Self-efficacy Scale<br>QoL (12-item)<br>Short Form Health Survey version 2—Chinese (HK)<br>Modified Barthel Index—Chinese version<br>The Lawton Instrumental Activities of Daily Living scale - Chinese version<br>Adherence to Refills and Medications Scale
20	Prevention	Physical and cognitive activities<br>Tai Chi-inspired exercises<br>Dancing<br>Step-based cognitive games	Accelerometer and gyroscope assessments<br>Short Physical Performance Battery (SPPB)<br>Senior Fitness Test (SFT)<br>Victoria Stroop Test (VST)<br>Trail Making Test (TMT)<br>Wechsler Memory Scale-Revised<br>MRI (Image) Acquisition
21	Supportive Care	Engage and empower to take action on preventing diseases<br>Maintaining and promoting health and functioning<br>Resolve their health and social problems proactively<br>Building up their self-care confidence<br>Providing referral services to other health care disciplines in the community	Health service utilization (Number of public GP visits, Number of private GP visits, Number of A&E admissions, Number of in-patient admissions, Total length of stay in hospital)<br>The 12-item Short-Form Health Survey (SF-12<br>HK)

22	Prevention	Physical activity<br>Productivity<br>Social connection<br>Medical<br>Maintenance of health<br>Diet and nutrition<br>Rehabilitation<br>Mental health<br>Smoking and use of alcohol<br>Other goals	10-item DSSI<br>Zarit Burden Interview<br>International Physical Activity Questionnaire (IPAQ)<br>General Self-Efficacy Scale (GSE)<br>EQ-5D
23	-	Concept of ageing<br>Healthy eating<br>Physical activity<br>Stress management<br>Taking responsibility for one's health<br>Interpersonal relationship<br>Spiritual growth<br>Exercise program	Participant's socio-demographic information<br>Health Promoting Lifestyle Profile II (HPLP II)
24	Prevention	Goals and needs<br>Resources<br>Personal qualities<br>Enabling<br>Group work skills	36 Item Short Form Health Survey (SF-36)<br>Patient Health Questionnaire (PHQ)<br>EQ-5D-3 L<br>de Jong Gierveld Loneliness Scale<br>General Self-Efficacy Scale (GSE)<br>Office for National Statistics (ONS) well-being<br>Serious adverse events (SAEs)<br>All health and social care use
25	Prevention	Exercise<br>Diet/nutrition<br>Cognitive activity to promote the health	Health Literacy Scale (HLS)-14<br>Food intake<br>Life-Space Assessment<br>Lubben Social Network Scale<br>Geriatric Depression Scale<br>Portable grip strength dynamometer<br>5-m walking test<br>Triaxial accelerometer<br>Demographic and Clinical Characteristics

2 Supplementary Material B.

	**Pubmed queries**	
Search	Query	Items found
#4	(("care"[All Fields] OR "take care"[All Fields] OR ("self care"[MeSH Terms] OR ("self"[All Fields] AND "care"[All Fields]) OR "self care"[All Fields] OR "selfcare"[All Fields]) OR "care of the self"[All Fields] OR ("care s"[All Fields] OR "cared"[All Fields] OR "carefulness"[All Fields] OR "cares"[All Fields] OR "empathy"[MeSH Terms] OR "empathy"[All Fields] OR "caring"[All Fields])) AND ("healthy ageing"[All Fields] OR "healthy aging"[All Fields]) AND ("older age"[All Fields] OR "older adults"[All Fields] OR "older people"[All Fields])) AND ((english[Filter] OR spanish[Filter]) AND (2016:2021[pdat]))	838
#3	(("self-care"[Title] OR "care"[Title]) AND "healthy"[Title] AND ("aging"[Title] OR "old age"[Title] OR "older adults"[Title])) AND ((english[Filter] OR spanish[Filter]) AND (2016:2021[pdat]))	10
#2	(("Self-care"[All Fields] OR "care"[All Fields]) AND "healthy"[All Fields] AND "older people"[All Fields]) AND ((english[Filter] OR spanish[Filter]) AND (2016:2021[pdat]))	627
#1	((care[Title/Abstract] OR "take care"[Title/Abstract] OR selfcare [Title/Abstract] OR "care of the self" [Title/Abstract] OR "be cared for"[Title/Abstract]) AND ("healthy ageing"[Title/Abstract] OR "healthy aging"[Title/Abstract])) AND ("older age") AND ((english[Filter] OR spanish[Filter]) AND (2016:2021[pdat]))	261

	**WoS queries**	
Search	Query	Items found
#6	ALL=((care OR “take care” OR selfcare OR “care of the self” OR “be cared for”) AND (“healthy ageing” OR “healthy	874

	aging”) AND (“older age” OR “older adults” OR “older people”))	
#5	ALL=((care OR selfcare) AND healthy AND (“old age” OR “older adults” OR aging))	4235
#4	ALL=((care OR “take care” OR selfcare OR “care of the self” OR “be cared for”) AND (“healthy ageing” OR “healthy aging”) AND (“older age” OR “older adults” OR “older people”) AND (intervention OR "RCT"))	5642
#3	TS=((care OR “take care” OR selfcare OR “care of the self” OR “be cared for”) AND (“healthy ageing” OR “healthy aging”) AND (“older age” OR “older adults” OR “older people”) AND (intervention OR "RCT") NOT disease)	89
#2	TS=((care OR “take care” OR selfcare OR “care of the self” OR “be cared for”) AND (“healthy ageing” OR “healthy aging”) AND (“older age” OR “older adults” OR “older people”) AND (intervention OR "RCT"))	181
#1	((TS=(care OR “take care” OR selfcare OR “care of the self” OR “be cared for”)) AND TS=(“healthy ageing” OR “healthy aging”)) AND TS=(“older age” OR “older adults” OR “older people”))	667

	**Scopus queries**	
Search	Query	Items found
#8	(ALL (care OR "take care" OR selfcare OR "care of the self" OR "be cared for") AND ALL ("healthy ageing" OR "healthy aging") AND ALL ("older age" OR "older adults" OR "older people")) AND (LIMIT-TO (PUBYEAR , 2021) OR LIMIT-TO (PUBYEAR , 2020) OR LIMIT-TO (PUBYEAR , 2019) OR LIMIT-TO (PUBYEAR , 2018) OR LIMIT-TO (PUBYEAR , 2017) OR LIMIT-TO (PUBYEAR , 2016)) AND (LIMIT-TO (LANGUAGE , "English") OR LIMIT-TO (LANGUAGE , "Spanish"))	14496
#7	(ALL (care OR "take care" OR selfcare OR "care of the self" OR "be cared for") AND ALL ("healthy ageing" OR "healthy	9126

	aging") AND ALL ("older age" OR "older adults" OR "older people") AND ALL (intervention OR "RCT")) AND (LIMIT-TO (PUBYEAR , 2021) OR LIMIT-TO (PUBYEAR , 2020) OR LIMIT-TO (PUBYEAR , 2019) OR LIMIT-TO (PUBYEAR , 2018) OR LIMIT-TO (PUBYEAR , 2017) OR LIMIT-TO (PUBYEAR , 2016)) AND (LIMIT-TO (LANGUAGE , "English") OR LIMIT-TO (LANGUAGE , "Spanish"))	
#6	(ALL (care OR "take care" OR selfcare OR "care of the self" OR "be cared for") AND ALL ("healthy ageing" OR "healthy aging") AND ALL ("older age" OR "older adults" OR "older people") AND NOT ALL (disease OR illness)) AND (LIMIT-TO (PUBYEAR , 2021) OR LIMIT-TO (PUBYEAR , 2020) OR LIMIT-TO (PUBYEAR , 2019) OR LIMIT-TO (PUBYEAR , 2018) OR LIMIT-TO (PUBYEAR , 2017) OR LIMIT-TO (PUBYEAR , 2016)) AND (LIMIT-TO (LANGUAGE , "English") OR LIMIT-TO (LANGUAGE , "Spanish"))	2536
#5	(ALL (care OR "take care" OR selfcare OR "care of the self" OR "be cared for") AND ALL ("healthy ageing" OR "healthy aging") AND ALL ("older age" OR "older adults" OR "older people") AND ALL (intervention OR "RCT") AND NOT ALL (disease OR illness)) AND (LIMIT-TO (PUBYEAR , 2021) OR LIMIT-TO (PUBYEAR , 2020) OR LIMIT-TO (PUBYEAR , 2019) OR LIMIT-TO (PUBYEAR , 2018) OR LIMIT-TO (PUBYEAR , 2017) OR LIMIT-TO (PUBYEAR , 2016)) AND (LIMIT-TO (LANGUAGE , "English") OR LIMIT-TO (LANGUAGE , "Spanish"))	1234
#4	(TITLE-ABS-KEY (care OR selfcare) AND TITLE-ABS-KEY (healthy) AND TITLE-ABS-KEY ("old age" OR "older adults" OR aging)) AND (LIMIT-TO (PUBYEAR , 2021) OR LIMIT-TO (PUBYEAR , 2020) OR LIMIT-TO (PUBYEAR , 2019) OR LIMIT-TO (PUBYEAR , 2018) OR LIMIT-TO (PUBYEAR , 2017) OR LIMIT-TO (PUBYEAR , 2016)) AND (LIMIT-TO (LANGUAGE , "English") OR LIMIT-TO (LANGUAGE , "Spanish"))	3367
#3	(TITLE-ABS-KEY (care OR "take care" OR selfcare OR "care of the self" OR "be cared for") AND TITLE-ABS-KEY ("healthy ageing" OR "healthy aging") AND TITLE-ABS-KEY ("older age" OR "older adults" OR "older people") AND TITLE-ABS-KEY (intervention OR "RCT") AND NOT TITLE-ABS-KEY (disease OR illness)) AND (LIMIT-TO (PUBYEAR , 2021) OR LIMIT-TO (	157

	PUBYEAR , 2020) OR LIMIT-TO (PUBYEAR , 2019) OR LIMIT-TO (PUBYEAR , 2018) OR LIMIT-TO (PUBYEAR , 2017) OR LIMIT-TO (PUBYEAR , 2016)) AND (LIMIT-TO (LANGUAGE , "English") OR LIMIT-TO (LANGUAGE , "Spanish"))	
#2	(TITLE-ABS-KEY (care OR "take care" OR selfcare OR "care of the self" OR "be cared for") AND TITLE-ABS-KEY ("healthy ageing" OR "healthy aging") AND TITLE-ABS-KEY ("older age" OR "older adults" OR "older people") AND TITLE-ABS-KEY (intervention OR "RCT")) AND (LIMIT-TO (PUBYEAR , 2021) OR LIMIT-TO (PUBYEAR , 2020) OR LIMIT-TO (PUBYEAR , 2019) OR LIMIT-TO (PUBYEAR , 2018) OR LIMIT-TO (PUBYEAR , 2017) OR LIMIT-TO (PUBYEAR , 2016)) AND (LIMIT-TO (LANGUAGE , "English") OR LIMIT-TO (LANGUAGE , "Spanish"))	238
#1	(TITLE-ABS-KEY (care OR "take care" OR selfcare OR "care of the self" OR "be cared for") AND TITLE-ABS-KEY ("healthy ageing" OR "healthy aging") AND TITLE-ABS-KEY ("older age" OR "older adults" OR "older people")) AND (LIMIT-TO (PUBYEAR , 2021) OR LIMIT-TO (PUBYEAR , 2020) OR LIMIT-TO (PUBYEAR , 2019) OR LIMIT-TO (PUBYEAR , 2018) OR LIMIT-TO (PUBYEAR , 2017) OR LIMIT-TO (PUBYEAR , 2016)) AND (LIMIT-TO (LANGUAGE , "English") OR LIMIT-TO (LANGUAGE , "Spanish"))	648

	**Medline queries**	
Search	Query	Items found
#6	TX (care OR selfcare) AND (healthy) AND (“old age” OR “older adults” OR aging) Limiters - Date of Publication: 20160101-20211231; Age Related: Aged: 65+ years<br><br>Expanders - Apply equivalent subjects<br><br>Search modes - Boolean/Phrase	23838
#5	TX (care OR “take care” OR selfcare OR “care of the self” OR “be cared for”) AND (“healthy ageing” OR “healthy	4016

	aging") AND ("older age" OR "older adults" OR "older people") Limiters - Date of Publication: 20160101-20211231; Age Related: Aged: 65+ years<br><br>Expanders - Apply equivalent subjects<br><br>Search modes - Boolean/Phrase	
#4	AB (care OR "take care" OR selfcare OR "care of the self" OR "be cared for") AND ("healthy ageing" OR "healthy aging") AND ("older age" OR "older adults" OR "older people") AND (intervention OR "RCT") Limiters - Date of Publication: 20160101-20211231; Age Related: Aged: 65+ years<br><br>Expanders - Apply equivalent subjects<br><br>Search modes - Boolean/Phrase	49
#3	AB (care OR selfcare) AND (healthy) AND ("old age" OR "older adults" OR aging) Limiters - Date of Publication: 20160101-20211231; Age Related: Aged: 65+ years<br><br>Expanders - Apply equivalent subjects<br><br>Search modes - Boolean/Phrase	513
#2	AB (care OR "take care" OR selfcare OR "care of the self" OR "be cared for") AND AB ("healthy ageing" OR "healthy aging") AND AB ("older age" OR "older adults" OR "older people") Limiters - Date of Publication: 20160101-20211231; Age Related: Aged: 65+ years<br><br>Expanders - Apply equivalent subjects<br><br>Search modes - Boolean/Phrase	139
#1	AB (care OR "take care" OR selfcare OR "care of the self" OR "be cared for") AND AB ("healthy ageing" OR "healthy aging") AND AB ("older age" OR "older adults" OR "older people") Limiters - Date of Publication: 20160101-20211231<br><br>Expanders - Apply equivalent subjects<br><br>Search modes - Boolean/Phrase	280

	**Cochrane Library queries**	
Search	Query	Items found
#4	(care OR "take care" OR selfcare OR "care of the self" OR "be cared for") AND ("healthy ageing" OR "healthy aging") AND ("older age" OR "older adults" OR "older people") NOT (disease OR illness) in Full text - with publication date in the Cochrane Library Between Jan 2016 and Dec 2021, in Trials	104
#3	(care OR "take care" OR selfcare OR "care of the self" OR "be cared for") AND ("healthy ageing" OR "healthy aging") AND ("older age" OR "older adults" OR "older people") in Title Abstract Keyword - with publication date in the Cochrane Library Between Jan 2016 and Dec 2021, in Trials	93
#2	(care OR "take care" OR selfcare OR "care of the self" OR "be cared for") AND ("healthy ageing" OR "healthy aging") AND ("older age" OR "older adults" OR "older people") NOT (disease OR illness) in Title Abstract Keyword - with publication date in the Cochrane Library Between Jan 2016 and Dec 2021, in Trials	84
#1	(care OR "take care" OR selfcare OR "care of the self" OR "be cared for") AND ("healthy ageing" OR "healthy aging") AND ("older age" OR "older adults" OR "older people") in Full text - with publication date in the Cochrane Library Between Jan 2016 and Dec 2021	112
